# Validation of the *FAM19A4*/*mir124-2* DNA methylation test for both lavage- and brush-based self-samples to detect cervical (pre)cancer in HPV-positive women

**DOI:** 10.1016/j.ygyno.2016.02.012

**Published:** 2016-05

**Authors:** Lise M.A. De Strooper, Viola M.J. Verhoef, Johannes Berkhof, Albertus T. Hesselink, Helena M.E. de Bruin, Folkert J. van Kemenade, Remko P. Bosgraaf, Ruud L.M. Bekkers, Leon F.A.G. Massuger, Willem J.G. Melchers, Renske D.M. Steenbergen, Peter J.F. Snijders, Chris J.L.M. Meijer, Daniëlle A.M. Heideman

**Affiliations:** a Department of Pathology, VU University Medical Center, Amsterdam, The Netherlands; b Department of Epidemiology and Biostatistics, VU University Medical Center, Amsterdam, The Netherlands; c Self-Screen B.V., Amsterdam, The Netherlands; d Department of Pathology, Erasmus MC, University Medical Center Rotterdam, Rotterdam, The Netherlands; e Department of Obstetrics and Gynaecology, Radboud University Medical Center, Nijmegen, The Netherlands; f Department of Medical Microbiology, Radboud University Medical Center, Nijmegen, The Netherlands

**Keywords:** Human papillomavirus, Self-sampling, Reflex test, Cervical cancer, Molecular screening, DNA methylation, HPV16/18 genotyping

## Abstract

**Objectives:**

DNA methylation analysis of cancer-related genes is a promising tool for HPV-positive women to identify those with cervical (pre)cancer (CIN3+) in need of treatment. However, clinical performance of methylation markers can be influenced by the sample type utilized. We describe a multiplex quantitative methylation-specific PCR that targets *FAM19A4* and *mir124-2* loci, to detect CIN3+ using both HPV-positive lavage- and brush self-samples.

**Methods:**

We determined methylation thresholds for clinical classification using HPV-positive training sets comprising lavage self-samples of 182 women (including 40 with CIN3+) and brush self-samples of 224 women (including 61 with CIN3+). Subsequently, independent HPV-positive validation sets of 389 lavage self-samples (including 78 with CIN3+), and 254 brush self-samples (including 72 with CIN3+) were tested using the preset thresholds. Furthermore, the clinical performance of combined methylation analysis and HPV16/18 genotyping was determined.

**Results:**

Training set analysis revealed similar *FAM19A4* and *mir124-2* thresholds for both self-sample types to yield highest CIN3+ sensitivity at 70% specificity. Validation set analysis resulted in a CIN3+ sensitivity of 70.5% (95%CI: 60.4–80.6) at a specificity of 67.8% (95%CI: 62.7–73.0) for lavage self-samples, and a CIN3+ sensitivity of 69.4% (95%CI: 58.8–80.1) at a 76.4% (95%CI: 70.2–82.6) specificity for brush self-samples. In combination with HPV16/18 genotyping, CIN3+ sensitivity and specificity were 88.5% (95%CI: 81.4–95.6) and 46.0% (95%CI: 40.4–51.5) for lavage self-samples, and 84.7% (95%CI: 76.4–93.0) and 54.9% (95%CI: 47.7–62.2) for brush self-samples.

**Conclusions:**

*FAM19A4*/*mir124-2* methylation analysis performs equally well in HPV-positive lavage- and brush self-samples to identify women with CIN3+. In combination with HPV16/18 genotyping, significantly higher CIN3+ sensitivities are obtained, at decreased specificity.

## Highlights

• *FAM19A4*/*mir124-2* triage of HPV-positive self-samples is a feasible and promising tool
• *FAM19A4*/*mir124-2* analysis identifies HPV-positive women at risk of cervical cancer.
• *FAM19A4*/*mir124-2* analysis performs equally well in lavage and brush self-samples.
• Further validation of HPV self-sampling with *FAM19A4*/*mir124-2* triage is warranted

## 1 Background

A substantial subset of women does not participate in population-based cervical screening, which compromises the effectiveness of the screening program [1]. These non-responders are at increased risk of developing cervical cancer, indicating the need for recruitment of these women into screening [1], [2]. Offering devices for self-collection of (cervico-)vaginal specimens has prospects to increase screening compliance [3], [4], [5], [6]. Self-collected (cervico-)vaginal specimens can be tested for the presence of DNA from high-risk types of human papillomavirus (HPV) in a laboratory (i.e., HPV self-sampling). When using validated PCR assays, HPV testing on self-samples can have similar accuracy for detecting cervical (pre)cancer (CIN3 +) as HPV testing of physician-taken samples [4], [7], [8], [9], [10], [11], [12]. Therefore, it is expected that HPV self-sampling with validated combinations of self-collection devices and HPV tests, will be increasingly adopted in future HPV-based screening programs [10], [13], [14].

However, HPV testing cannot distinguish transient from persistent, clinically relevant high-risk HPV infections. Therefore, additional testing (i.e., triage) is essential to identify HPV-positive women with CIN lesions in need of treatment. This approach will reduce over-referral, unnecessary colposcopies, and overtreatment of women without clinically meaningful HPV infections. Currently, various triage strategies for hrHPV-positive women have been considered including repeat cytology testing [15], HPV16/18 genotyping [16], [17] (and combinations thereof [15], [18]), HPV E7 mRNA analysis [19], [20], viral and/or host cell DNA methylation analysis [21], [22], [23], p16/ki67 cytological dual staining [24], [25] and analysis of host cell gene amplification such as *TERC* [26], [27]. Of these, cytology, either or not combined with HPV16/18 genotyping analysis, is currently most widely accepted [15], [28]. However, cytology on self-collected (cervico-)vaginal specimens is unreliable [29]. Therefore, cytology triage would require an additional visit to a clinician for cervical scrape collection. The resulting prerequisite for cervical cytology for previous non-responder women, has been associated with loss to follow-up and has shown to extend the diagnostic track [22]. This process could be simplified substantially by triage testing directly on HPV-positive self-samples by non-morphological, molecular methods.

Although HPV16/18 genotyping as molecular test is directly applicable to self-samples and detects women with increased cervical cancer risk, a non-negligible fraction of (pre)cancers associated with non-HPV16/18 high-risk HPV types will be missed when using genotyping alone [30]. As an alternative or additive tool, methylation markers that reflect molecular events in host cells contributing to cervical carcinogenesis are highly promising [31], [32], [33]. Previous studies have revealed that promoter methylation of host cell genes such as *FAM19A4* and *mir124-2*, increases with cervical disease severity [34], [35], [36]. Methylation levels of *FAM19A4* and *mir124-2* are particularly high in women with cervical cancer and advanced high-grade lesions, the latter characterized by a longer duration (≥ 5 years) of a preceding high-risk HPV infection [31], [35], [37], [38]. Of interest, methylation analysis on HPV-positive self-collected lavage samples as direct molecular triage tool using *MAL* and *mir124-2* loci has reported to be clinically non-inferior to cytology triage on a subsequent physician-taken scrape in a recent randomized controlled trial [22]. In addition, methylation analysis on these samples could be combined with HPV16/18 genotyping to reach higher CIN3+ sensitivity [30].

With an increasing interest in self-collection for HPV-based cervical screening programs [39], [40], it is of importance to gain more clinical performance data on methylation marker analysis of HPV-positive self-samples. Since performance of methylation markers can be influenced by the type of sample utilized [41], performance evaluation in different self-sample types is necessary to determine its broader utility. Unlike samples collected by lavage-based device [22], [33], evaluation of brush-collected self-samples by methylation analysis is restricted to feasibility studies so far [32]. Furthermore, no studies with large sample numbers have compared the performance of DNA methylation markers on HPV-positive lavage and brush self-samples.

Here, we conducted a post-hoc analysis on 1049 HPV-positive self-samples from previous PROHTECT studies that had been collected by either lavage- and or brush-based self-collection devices [5], [22], [42]. We evaluated a multiplex quantitative methylation-specific PCR (qMSP) that targets *FAM19A4* and *mir124-2* loci. The performance of the qMSP assay in terms of analytical sensitivity, analytical specificity, and daily-use reproducibility is described. In addition, the clinical performance of the assay, either or not combined with HPV16/18 genotyping, for detection of cervical (pre)cancer was determined on both self-sample types.

## 2 Methods

### 2.1 Study populations

#### 2.1.1 Lavage-collected self-samples

**Fig. 1:**
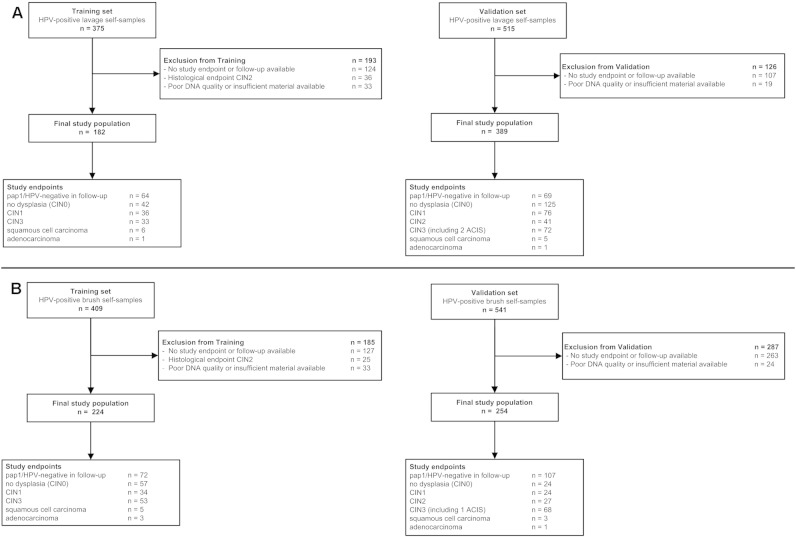
Overview of the study populations. The training and validation sets for lavage self-samples (1A), and the training and validation sets for brush self-samples (1B) are presented. HPV = human papillomavirus, CIN = cervical intraepithelial neoplasia.

For the training set, 375 HPV-positive (GP5 +/6 + PCR) cervico-vaginal lavage self-samples collected with the second generation Delphi-screener (Delphi-Bioscience, the Netherlands) from non-responder women participating in the PROHTECT-3B trial were available. The flowchart of this training set is shown in Fig. 1A (left panel). These samples are further referred to as lavage self-samples. The trial was registered in the trial register as NTR3350. Detailed characteristics, inclusion criteria and follow-up procedures of the PROHTECT-3B trial have been described previously [42]. Of the HPV-positive women, 15 women had no cytological or histological follow-up and were excluded from analysis. Of the remaining 360 women, 251 women reached a study-endpoint [i.e., either a histological outcome, classified as cervical cancer, cervical intraepithelial neoplasia (CIN) grade 3 (CIN3), CIN2, CIN1, or absence of CIN (CIN0) or a combined normal cytology and HPV negative outcome]. *S*ince CIN2 lesions often represent a misclassified CIN1 or CIN3, women with CIN2 lesions (*n* = 36) were excluded from the training set. From the remaining 215 women, 182 had sufficient quantities of the self-sample with qualitatively adequate DNA left for qMSP analysis. Of them, 40 were histologically diagnosed with CIN3+ [i.e., 33 CIN3, 6 squamous cell carcinoma (SCC), and 1 adenocarcinoma (AdCA)] with a median age of 38 years (range 33–58). The remaining 142 women had no evidence of CIN2 + (also referred to as women with ≤ CIN1). Of these, 42 had histologically-confirmed absence of CIN, 36 had CIN1 and 64 women had both a negative HPV test and normal cytology at follow-up The median age of women with ≤ CIN1 was 38 years (range 33–64).

For the validation set, 515 HPV-positive (GP5 +/6 + − PCR) lavage self-samples, collected with the first generation Delphi-screener (Delphi-Bioscience, the Netherlands) were available from non-responder women participating in the methylation triage arm of the PROHTECT-3A trial (NTR2606). The first and second generation Delphi-screener perform equal in DNA yield and HPV-detection [43]. The flowchart of the validation set is shown in Fig. 1A (right panel). Detailed characteristics, inclusion criteria and follow-up procedures of the PROHTECT-3A trial have been described previously [22]. Of the 515 HPV-positive women, 408 women reached a study-endpoint (as detailed above) within 18 months of follow-up. Of the 408 women, 389 had sufficient quantities left of the self-sample with qualitatively adequate DNA for qMSP analysis. This series included 78 women who were histologically diagnosed with CIN3+ [i.e., 70 CIN3, 5 SCC, 2 adenocarcinoma in situ (ACIS) and 1 AdCA] with a median age of 38 years (range 33–58) and 41 women with CIN2 with a median age of 43 years (range 33–58). The remaining 270 women had no evidence of CIN2 +. Of these, 125 had histologically-confirmed absence of CIN and 76 had CIN1. The remaining 69 women had both a negative HPV test and normal cytology at follow-up. The median age of this group was 43 years (range 33–63).

#### 2.1.2 Brush-collected self-samples

For the training set, 409 HPV-positive (GP5 +/6 + PCR) vaginal brush self-samples collected with the Evalyn brush (Rovers Medical Devices, the Netherlands) from non-responder women participating in the PROHTECT-3B trial [42] were available. The flowchart of this training set is shown in Fig. 1B (left panel). After self-sampling, the brushes were sent dry to the laboratory where they were placed into 1.5 ml PreservCyt medium (Hologic, USA) before further processing. Of the HPV-positive women, 18 women had no cytological or histological follow-up and were excluded from analysis. From the 391 HPV-positive women, those who reached a study-endpoint (as detailed above), except for those with CIN2 (*n* = 25, as explained above), and of whom sufficient quantities were left of the self-sample with qualitatively adequate DNA for qMSP analysis, were included in the training set. The remaining series of 224 women included 61 women who were histologically diagnosed with a CIN3+ lesion (i.e., 53 CIN3, 5 SCC, and 3 AdCA) with a median age of 38 years (range 33–59) The other 163 women had no evidence of CIN2 +. Of these, 57 had histologically-confirmed absence of CIN, 34 had CIN1, and 72 women had both a negative HPV test and normal cytology at follow-up The median age of women with ≤ CIN1 was 38 years (range 33–63).

For the validation set, 541 HPV-positive (GP5 +/6 + PCR) vaginal brush self-samples from non-responder women participating in the PROHTECT-2 trial (NTR1851), were available. These samples were collected with the VibaBrush (Rovers Medical Devices, the Netherlands). The flowchart of the validation set is shown in Fig. 1B (right panel). After self-sampling, the brushes were placed in 1.5 ml universal collection medium (Qiagen, USA) before sending to the laboratory. Detailed characteristics, inclusion criteria and follow-up procedures of the PROHTECT-2 trial have been described previously [7]. From the 541 HPV-positive women, only those who reached a study-endpoint (as detailed above) within 36 months of follow-up, and of whom sufficient quantities were left of the self-sample with qualitatively adequate DNA for qMSP analysis, were included in the validation set (*n* = 254). Of these, 72 women were histologically diagnosed with a CIN3+ lesion [i.e., 67 CIN3, 3 SCC, 1 ACIS and 1 AdCA] and had a median age of 36 years (range 31–61). 27 women were diagnosed with CIN2 and had a median age of 36 years (range 31–56). The remaining 155 women had no evidence of CIN2 +. Of these women, 24 had histologically-confirmed absence of CIN, 24 had CIN1, and 107 women had both a negative HPV test and normal cytology at follow-up. The median age of this group was 36 years (range 30–62).

All PROHTECT trials had ethical approval by the National Health Council and all participants gave informed consent.

### 2.2 Cytology and histology

Women with an HPV-positive self-sample were referred for a colposcopy-directed biopsy in case of a positive triage test at baseline. In case of a negative triage test, HPV-positive women were invited for a repeat co-test (HPV and cytology) after 6–12 months. If at least one of these tests was abnormal (i.e. abnormal cytology (≥ borderline or mild dyskaryosis) or hrHPV-positive), women were referred for colposcopy-directed biopsy as well. Biopsies taken at colposcopy were histologically assessed in participating hospitals and classified as normal (CIN0), CIN1, CIN2, CIN3 or invasive cancer, according to international criteria [44]. Women were treated according to standard procedures in the Netherlands. Cervical scrapes were classified according to the CISOE-A classification (reporting on Composition, Inflammation, Squamous, Other and endometrium, Endocervical cylindrical epithelium, and Adequacy) used in the Netherlands [45]. The results can be translated into the Bethesda classification [46] in which borderline or mild dyskaryosis (BMD) equals ASC-US/ASC-H/LSIL, and > BMD equals high-grade squamous intraepithelial lesion (HSIL).

#### 2.2.1 DNA isolation and HPV genotyping

DNA from lavage and brush self-samples was isolated using the Nucleo-Mag 96 Tissue kit (Macherey-Nagel, Germany) and Microlab Star robotic system (Hamilton, Germany) according to manufacturers' protocol [47]. In the PROHTECT-3 trials, all samples were tested for high-risk HPV (hrHPV) DNA by the clinically validated hrHPV GP5 +/6 + PCR (EIA HPV GP HR kit, Diassay, The Netherlands) according to the manufacturer's protocol [48]. In PROHTECT-3A, subsequent hrHPV genotyping was performed using Luminex suspension array technology [49]. In the PROHTECT-2 trial, samples were tested for hrHPV using the Hybrid Capture-2® (HC2, Qiagen, USA) according to manufacturer's protocol [50]. All HC2 positive samples were subsequently tested by GP5 +/6 + PCR and subjected to hrHPV genotyping by the reverse line blot assay [51]. In the current study, only samples that were GP5 +/6 + PCR-positive were used.

#### 2.2.2 Bisulphite treatment and qMSP methylation analysis

Isolated DNA was subjected to bisulphite treatment using the EZ DNA Methylation Kit (Zymo Research, USA) as described previously [52], [53]. Bisulphite-converted DNA (50 ng) was used as template for DNA methylation analysis. DNA methylation analysis was performed by a multiplex qMSP assay targeting *FAM19A4* and *mir124-2* loci, as well as *ACTB* as a sample quality control. Analyses were performed on an ABI 7500 real-time PCR-system (Applied Biosystems, USA). For each target, Quantification Cycle (Cq) values were measured at a fixed fluorescence threshold. The result of a sample is expressed in ΔΔCq ratio being a measure for hypermethylation using the 2^-ΔΔCq^ method [54]. A plasmid containing all amplimer sequences (i.e., *ACTB*, *FAM19A4*, and *mir124-2*) was used as amplification target to assess the analytical sensitivity. Serial 10-fold plasmid dilutions (range from 750,000 to 1 copy per reaction) were analysed in duplicate in two independent qMSP runs. The analytical sensitivity was determined as the lowest dilution giving 4 out of 4 positive results (Cq < 40). To assess the analytical specificity, bisulphite-converted unmethylated DNA from primary keratinocytes and unmodified DNA (i.e., pool of non-bisulphite treated DNA from cervical samples) were analysed in quadruplicate. The reproducibility of the assay based on ΔΔCq values was evaluated by duplicate measurement in independent qMSP runs of bisulphite-converted DNA of 30 HPV-positive cervical samples.

#### 2.2.3 Data and statistical analysis

First, both training sets (lavage self-samples and brush self-samples, separately) were used to define clinical decision points, i.e., thresholds for positivity of the triage assay. At a predefined specificity value of 70% for CIN3+, an optimization procedure was performed to calculate the maximum corresponding CIN3+ sensitivity over all possible threshold values for the combined marker panel. The defined methylation thresholds converted the test result into a categorical variable leading to an optimal CIN3+ sensitivity at 70% CIN3+ specificity level. The thresholds were subsequently evaluated in the two independent validation sets (lavage and brush, respectively). The primary outcome was CIN3+ detection and the secondary outcome was CIN2 + detection. In the validation sets, all samples were additionally evaluated for the clinical performance of *FAM19A4*/*mir124-2* methylation analysis combined with HPV16/18 genotyping. Sensitivity, specificity, positive predictive value (PPV), and negative predictive value (NPV) were determined for outcome CIN3+ and CIN2+ with 95% Wald confidence intervals. The colposcopy referral rate was calculated as the proportion of HPV-positive women who had a positive triage test result. Analyses were performed in the R package (version 2.15). Additional calculations were performed in IBM SPSS Statistics 20 and Excel.

## 3 Results

### 3.1 Analytical performance

**Fig. 2:**
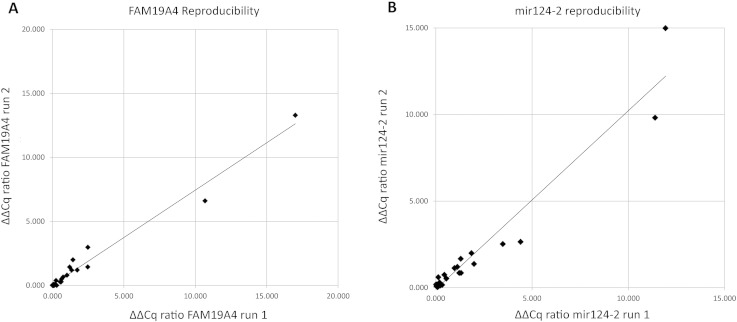
Reproducibility of the ΔΔcq ratios of *FAM19A4* (A) and *mir124-2* (B) between two independent qMSP runs.

For assessing DNA methylation of *FAM19A4* and *mir124-2*, a multiplex qMSP assay was used. The analytical sensitivity of the assay was 2.5 copies of methylated target per PCR reaction for both *FAM19A4* and *mir124-2*, and the reference gene *ACTB*. The assay revealed no positive signals with bisulphite-converted unmethylated DNA nor non-bisulphite treated DNA of cervical samples, showing a high analytical specificity. The assay demonstrated a high reproducibility on HPV-positive cervical samples with Pearson correlation coefficients between the ΔΔCq values of 0.988 for *FAM19A4* (Fig. 2A) and 0.970 for *mir124-2* (Fig. 2B).

### 3.2 Clinical performance

Using the training sets of HPV-positive lavage and brush self-samples separately, methylation thresholds for *FAM19A4* and *mir124-2* were determined that resulted in a maximum CIN3+ sensitivity at a preset specificity of 70%. Of interest, these thresholds did not differ between the different sample types (i.e., HPV-positive lavage compared to brush self-samples). According to the thresholds, samples with ΔΔCq ratios above the respective threshold for at least one of the targets (*FAM19A4* and/or *mir124-2*) were rated as test-positive, while samples with ΔΔCq ratio of both targets below their threshold were rated as test-negative. The use of these thresholds resulted in CIN3+ sensitivities of 75.0% (95%CI: 61.6–88.4) and 72.1% (95%CI: 60.9–83.4) in the training sets of HPV-positive lavage self-samples and brush self-samples, respectively.

Next, the clinical performance of the multiplex qMSP assay was validated on independent sets of HPV-positive lavage and brush self-samples. Sensitivities, specificities, PPV, NPV and referral rate for colposcopy for endpoints CIN3+ and CIN2+ are presented in Table 1 and Supplementary Table 1, respectively. At the predefined threshold of 70%, a CIN3+ sensitivity of 70.5% (95%CI: 60.4–80.6) at a specificity of 67.8% (95%CI: 62.7–73.0) was obtained for HPV-positive lavage self-samples. For CIN2+, a sensitivity of 63.9% (95%CI: 55.2–72.5) at 70.7% (95%CI: 65.3–76.2) specificity was obtained. In the validation set of HPV-positive brush self-samples, a CIN3+ sensitivity of 69.4% (95%CI: 58.8–80.1) at a specificity of 76.4% (95%CI: 70.2–82.5), and a CIN2+ sensitivity of 59.6% (95%CI: 49.9–69.3) at 78.1 (95%CI: 71.5–84.5) specificity was obtained. Of note, all women with cervical carcinomas (lavage, *n* = 13; brush, *n* = 12) were scored methylation positive on their self-sample by the *FAM19A4*/*mir124-2* methylation marker assay at the predefined 70% specificity threshold.

After combining *FAM19A4*/*mir124-2* methylation analysis with HPV16/18 genotyping, CIN3 + sensitivities increased and specificities decreased for both self-sample types. In lavage self-samples, a CIN3 + sensitivity of 88.5% (95%CI: 81.4–95.6) at a specificity of 46.0% (95%CI: 40.4–51.5) was observed. In brush self-samples, a CIN3 + sensitivity of 84.7% (95%CI: 76.4–93.0) at a specificity of 54.9% (95%CI: 47.7–62.2) was observed (Table 1). For CIN2 +, a similar tendency is seen (Supplementary Table 1).

## 4 Discussion

In this study, we evaluated *FAM19A4*/*mir124-*2 methylation analysis for lavage- and brush-based self-samples to detect cervical (pre)cancer in HPV-positive women. In comparison to the *MAL*/*mir124-2* triage test that was prospectively evaluated in HPV-positive lavage self-samples in a screening setting [22], the current assay shows a similar CIN3+ sensitivity at a higher specificity. Since the *FAM19A4*/*mir124-2* assay features likewise clinical performance in HPV-positive brush-collected self-samples as well as lavage-collected self-samples, it can be considered as a promising, universal triage test for HPV-positive (cervico-)vaginal self-samples collected by different self-collection devices. In combination with HPV16/18 genotyping, significantly higher sensitivities were obtained, yet at the cost of decreased specificities. Our study findings support further validation of cervical screening studies with HPV testing combined with triage by *FAM19A4*/*mir124-2* methylation analysis on self-collected (cervico-)vaginal specimens. To our knowledge this study is the first to evaluate in large sample series whether DNA methylation analysis is equally applicable to both HPV-positive lavage- and brush-collected self-samples for CIN3+ detection. Several studies have described DNA methylation of promotor regions of genes as candidate biomarkers [55]. Although some are highly promising for future application in molecular cervical screening, not all markers perform well and only a limited number of DNA methylation markers have been studied extensively as triage tests for HPV-positive cervical scrapes and/or self-samples [22], [23], [47], [56]. Differences in clinical performance of methylation markers between sample types have been reported before [41]. These differences are most likely related to intrinsic underlying variability in cellular composition and proportion of hypermethylation-positive cervical cells indicative for CIN lesions between various types of samples. Regarding self-samples, only lavage samples have been extensively investigated by DNA methylation marker analysis so far. The *JAM3*/*EPB4*/*TERT*/*C13ORF18* marker panel showed good feasibility on lavage self-samples [33] and the *MAL*/*mir124-2* marker panel performed non-inferior to cytology triage via a physician-taken cervical scrape for the detection of CIN2+ [22], [41]. For brush samples, only a feasibility study evaluating *JAM3*/*EPB4*/*TERT*/*C13ORF18* methylation has been published [32], but no clinical performance data in large series have been reported. The equal clinical performance of *FAM19A4*/*mir124-2* methylation analysis in both HPV-positive lavage and brush self-samples as assessed on large sample series herein, suggests that this assay is an attractive, directly applicable molecular triage tool for self-samples, independent of the collection device used. Further prospective studies are warranted to clinically validate HPV testing combined with *FAM19A4*/*mir124-2* methylation-based triage on self-collected (cervico-)vaginal samples in cervical screening studies.

The qMSP assay used in this study allows fast and reliable read-out of multiple methylation markers and a reference gene in one assay. As such, the methylation test safes clinical material, time and costs and improves quality control. Using a dichotomized outcome, the assay provides a clinical decision point to refer an HPV-positive woman for colposcopy or not. The advantages of our study are the evaluation of large, independent series of different self-sample types, and the use of a standardized assay. A limitation of our study can be seen in the difference in follow-up time between the cohorts used in this study (i.e., PROHTECT-2: 36 months; PROHTECT-3A: 18 months). However, in the PROHTECT-2 cohort, the far majority of women (i.e., 69/72) women developed CIN3+ during the first 18 months of follow-up and only 3/72 women during the 2nd 18 months of follow-up. As the number of women diagnosed with CIN3+ during the 2nd 18 months in PROHTECT-2 is limited, we feel that this has no meaningful influence on the clinical performance figures presented in this work for both cohorts.

To the best of our knowledge, no other methodology is currently available that can be reliably applied directly to self-collected (cervico-)vaginal specimens, and has similar clinical sensitivity and clinical specificity figures for the triage of HPV-positive women following self-sampling as the *FAM19A4*/*mir124-2* assay described herein. Cytology is a widely accepted triage test for HPV-positive women, but cytology triage following HPV self-sampling requires an additional cervical scrape taken by a physician [22], [29]. Furthermore, it has been proposed that methylation analysis has a high detection sensitivity for cancer and advanced cervical lesions having a high short-term progression risk for cancer [35]. In contrast, cytology detects with a moderate sensitivity all morphological cellular abnormalities associated with most, but not all, CIN2/3 and cancer [31]. Indeed, methylation analysis has shown to detect all cervical carcinomas [35], [38], as confirmed herein for limited numbers (*n* = 13 and *n* = 12 for lavage and brush self-samples, respectively). Nonetheless, part of CIN2 and few CIN3 lesions are likely to remain undetected when a methylation marker-based triage strategy is used. Based on our previous work, these lesions are likely early-onset or incident lesions with a low progression-risk to invasive cancer [35]. As clinicians prefer to also detect these early-onset lesions, combined molecular triage by *FAM19A4*/*mir124-2* methylation marker analysis and HPV16/18 genotyping may be considered for triage of women with an HPV-positive self-sample [30]. Indeed, CIN3+ sensitivities increased when adding HPV16/18 genotyping to *FAM19A4*/*mir124-2* methylation analysis. These findings are in line with Verhoef et al. [30] showing that combined molecular triage by *MAL*/*mir124-2* and HPV16/18 genotyping on HPV-positive lavage self-samples leads to significantly higher sensitivities for CIN3+, yet at the cost of a lower specificity.

## 5 Conclusion

*FAM19A4*/*mir124-2* methylation analysis performs equally well in HPV-positive lavage- and brush self-samples to identify women with CIN3+. In combination with HPV16/18 genotyping, significantly higher CIN3+ sensitivities are obtained, at decreased specificity. Further validation of molecular cervical screening with self-sampling in population-based study is warranted.

The following is the supplementary data related to this article.

Supplementary Table 1 Clinical performance of *FAM19A4/mir124-2* methylation marker analysis, HPV16/18 genotyping and the combination of both triage tests for outcome CIN2+ in the validation sets stratified by self-sample type.

## Competing interests

CJLMM, PJFS, RDMS and DAMH have minority stake in Self-Screen B.V., a spin-off company of VU University Medical Center Amsterdam. ATH is an employee of Self-screen B.V. CJLMM has participated in the sponsored speaker's bureau of Merck, GSK, Qiagen, Menarini, Segeene, and Roche, and served occasionally on the scientific advisory board of GSK, Qiagen, Merck, and Roche. CJLMM has occasionally been consultant for Qiagen and Genticel and is a minority shareholder of Diassay B.V. Formerly CJLMM was a minority shareholder of Delphi Biosciences. PJFS has been on the speaker's bureau of Roche, Abbott, Gen-Probe, Qiagen and Seegene. He is consultant for Crucell Holland B.V. DAMH has been on the speaker's bureau of Hologic/Gen-Probe and serves occasionally on the scientific advisory boards of AMGEN and Pfizer. JB has played an advisory role for Merck and Roche and has been on the speakers' bureau of Qiagen. RLMB received grants from Roche and SP-MSD and has participated in the speakers' bureau of Roche. All other authors declare that they have no conflicts of interest.

## Figures and Tables

**Table 1:** Clinical performance of *FAM19A4*/*mir124-2* methylation marker analysis, HPV16/18 genotyping and the combination of both triage tests for outcome CIN3+ in the validation sets stratified by self-sample type.

Self-sample	Triage marker	n1/N1	Sensitivity (%)	(95%CI)	n2/N2	Specificity (%)	(95%CI)	PPV (%)	(95%CI)	NPV (%)	(95%CI)	Referral rate
Lavage	*FAM19A4/mir124-2* methylation	55/78	70.5	(60.4–80.6)	211/311	67.8	(62.7–73.0)	35.5	(28.0–43.0)	90.2	(86.4–94.0)	39.8
Brush	*FAM19A4/mir124-2* methylation	50/72	69.4	(58.8–80.1)	139/182	76.4	(70.2–82.6)	53.8	(43.6–63.9)	86.3	(81.0–91.6)	36.6
Lavage	HPV16/18 genotyping	51/78	65.4	(54.8–75.9)	202/311	65.0	(59.7–70.3)	31.9	(24.7–39.1)	88.2	(84.0–92.4)	41.1
Brush	HPV16/18 genotyping	50/72	69.4	(58.8–80.1)	129/182	70.9	(64.3–77.5)	48.5	(38.9–58.2)	85.4	(79.8–91.1)	40.6
Lavage	*FAM19A4*/*mir124-2* methylation and/or HPV16/18 genotyping	69/78	88.5	(81.4–95.6)	168/311	46.0	(40.4–51.5)	29.1	(23.3–34.9)	94.1	(90.3–97.8)	60.9
Brush	*FAM19A4*/*mir124-2* methylation and/or HPV16/18 genotyping	61/72	84.7	(76.4–93.0)	100/182	54.9	(47.7–62.2)	42.7	(34.6–50.8)	90.1	(84.5–95.7)	56.3

CIN = cervical intraepithelial neoplasia; CI = confidence interval; PPV = positive predictive value; NPV = negative predictive value; n1 = number of test positive disease cases; N1 = total number of disease cases; n2 = number of test negative non-disease cases; N2 = total number of non-disease cases.
