# Supplementary material for: Validation of the FAM19A4/mir124-2 DNA methylation test for both lavage- and brush-based self-samples to detect cervical (pre)cancer in HPV-positive women
Source: Gynecol Oncol. 2016 May;141(2):341–7. doi: 10.1016/j.ygyno.2016.02.012 (PMC4851217; doi:10.1016/j.ygyno.2016.02.012)
Supplement: Supplementary Table 1 — Clinical performance of FAM19A4/mir124-2 methylation marker analysis, HPV16/18 genotyping and the combination of both triage tests for outcome CIN2+ in the validation sets stratified by self-sample type. [file mmc1.pdf]

SUPPLEMENTARY TABLE

Supplementary Table 1: Clinical performance of *FAM19A4/mir124-2* methylation marker analysis, HPV16/18 genotyping and the combination of both triage tests for outcome CIN2+ in the validation sets stratified by self-sample type.

Self-sample	Triage marker	n1 / N1	Sensitivity (%)	(95%CI)	n2 / N2	Specificity (%)	(95%CI)	PPV (%)	(95%CI)	NPV (%)	(95%CI)	Referral rate (%)
lavage	*FAM19A4/mir124-2* methylation	76 / 119	63.9	(55.2 - 72.5)	191 / 270	70.7	(65.3 - 76.2)	49.0	(41.2 - 56.9)	81.6	(76.7 - 86.6)	39.8
brush	*FAM19A4/mir124-2* methylation	59 / 99	59.6	(49.9 - 69.2)	121 / 155	78.1	(71.6 - 84.6)	63.4	(53.6 - 73.2)	75.2	(68.5 - 81.8)	36.6
lavage	HPV16/18 genotyping	67 / 119	56.3	(47.4 - 65.2)	177 / 270	65.6	(59.9 - 71.2)	41.9	(34.2 - 49.5)	77.3	(71.9 - 82.7)	41.1
brush	HPV16/18 genotyping	64 / 99	64.6	(55.2 - 74.1)	116 / 155	74.8	(68.0 - 81.7)	62.1	(52.8 - 71.5)	76.8	(70.1 - 83.6)	40.6
lavage	*FAM19A4/mir124-2* methylation and/or HPV16/18 genotyping	94 / 119	79.0	(71.7 - 86.3)	127 / 270	47.0	(41.1 - 53.0)	39.7	(33.4 - 45.9)	83.6	(77.7 - 89.4)	60.9
brush	*FAM19A4/mir124-2* methylation and/or HPV16/18 genotyping	78 / 99	78.8	(70.7 - 86.8)	90 / 155	58.1	(50.3 - 65.8)	54.5	(46.4 - 62.7)	81.1	(73.8 - 88.4)	56.3

CIN= cervical intraepithelial neoplasia; CI= confidence interval; PPV= positive predictive value; NPV= negative predictive value; n1= number of test positive disease cases; N1= total number of disease cases; n2= number of test negative non-disease cases; N2= total number of non-disease cases
